# Predicting Mortality and Life Expectancy in Patients under Prolonged Mechanical Ventilation and Maintenance Dialysis

**DOI:** 10.1089/jpm.2018.0646

**Published:** 2019-12-23

**Authors:** Chang-Sheng Jang, Jung-Der Wang

**Affiliations:** ^1^ Puli Christian Hospital, Nantou County, Taiwan.; ^2^ Department of Public Health, National Cheng Kung University College of Medicine, Tainan, Taiwan.; ^3^ Department of Internal Medicine, National Cheng Kung University Hospital, Tainan, Taiwan.; ^4^ Department of Occupational and Environmental Medicine, National Cheng Kung University Hospital, Tainan, Taiwan.

**Keywords:** end-of-life care, maintenance dialysis, mortality, palliative care, prolonged mechanical ventilation, survival

## Abstract

***Background:*** The number of patients receiving prolonged mechanical ventilation (PMV) or maintenance dialysis (MD) is increasing worldwide. Identification of those with a short life expectancy is useful for early referral of palliative care.

***Objective:*** To determine the survival rate, life expectancy, and major prognostic factors in patients under both PMV and MD.

***Design:*** We extracted a 1:3.4 random sample of patients treated with mechanical ventilation (MV) from the National Health Insurance (NHI) Research Database of Taiwan from 2003 to 2007. Subjects who had undergone MD and received MV for longer than 21 days were enrolled.

***Setting/Subjects:*** There were 1035 patients who received both PMV and MD.

***Measurements:*** The survival rates and life expectancy were estimated. A multivariate proportional hazards model was constructed to validate the effects of different prognostic factors, including age, gender, hospital size, and major comorbidities.

***Results:*** The median length of survival of patients under both PMV and MD was 54 days. The three-month, six-month, and one-year survival rates were 40.8%, 24.1%, and 12.6%, respectively. The life expectancies of those older than 70 years were five months; those comorbid with cancer and septicemia were 112 and 90 days, respectively. After adjustments for covariates, we found following prognostic factors were statistically significant: gangrene, peritonitis, liver cirrhosis, cancer, septicemia, hydrocephalus, having device complications, and shock.

***Conclusions:*** More than 85% of patients receiving both PMV and MD died within one year. Communication and early referral for palliative care would be indicated for those comorbid with significant prognostic factors.

## Introduction

The number of patients receiving prolonged mechanical ventilation (PMV) or maintenance dialysis (MD) due to end-stage renal disease has been consistently increasing as a result of aging population, multiple comorbidities, and the advances of life-sustaining technologies in critical care.^1–6^ These advances of life-sustaining technologies have enabled acutely and critically ill patients to survive, but not always recover, and thus created a growing population dependent on mechanical ventilation (MV) and other intensive care therapies, who have also been described as chronically critically ill (CCI) patients.^7^

PMV is defined as requiring more than 6 hours/day of MV for at least 21 consecutive days.^8^ For patients who are unable to wean from MV within three weeks of intubation, the next step would usually be for them to receive tracheostomy and then be transferred to a specialized respiratory care unit for further trials of weaning or long-term care. When making these medical decisions, the expected outcomes of patients receiving PMV are important. If such patients are in the terminal stage of their illness or the goal of weaning from MV seems too difficult to achieve, health care professionals might consider referral for palliative care.

Patients receiving PMV or MD are usually accompanied by increased mortality, morbidities, and health care cost, which would also affect their quality of life.^4,5,9–13^ Thus, it is crucial to understand their prognostic factors and make an early referral for palliative treatment.

A meta-analysis of patients receiving MV for >14 days found that fewer than half of patients survived beyond 1 year.^14^ Another systematic review of long-term mortality among patients receiving PMV, the authors identified six major prognostic factors, including advanced age, thrombocytopenia, acute kidney injury, vasopressor dependence, preexisting kidney injury, and failure to wean from MV.^15^ Moreover, research has indicated that almost 75% of patients receiving PMV in hospitals showed pain or discomfort and over 60% had poor cognition.^16^

Even among patients with fair to good cognition during hospital stays with PMV, over 80% were confined to bed and unable to maintain any self-care or usual activities. Thus, many CCI patients depend on family members or friends as surrogates for decision making. Unfortunately, research indicates that neither patients nor surrogates are well informed about long-term outcomes of being CCI.^17^ One study reported that physicians discussed with 26% of CCI surrogates about prognosis, functional limitations, quality of life, or expected caregiving needs, and there are big gaps on the anticipated prognosis between surrogates and physicians.^18^

There have been relatively few studies, however, focused on predicting the survival of patients receiving both PMV and MD. Most people prefer to die at home, yet most do not. Understanding factors associated with terminal hospitalization may inform interventions to improve care.^5^ Identifying the characteristics of those with short survival times may facilitate the early arrangement of family visits and palliative or end-of-life care. We thus conducted this study to determine the survival rate, life expectancy, and prognostic factors in patients receiving both PMV and MD.

## Methods

### Design, setting, and population

The study was approved by the Institutional Review Board of National Taiwan University Hospital (IRB200912072R). The data were retrieved from the National Health Insurance (NHI) Research Database, a reimbursement data file that was obtained from the NHI of Taiwan and was linked with the National Mortality Registry to ascertain mortality. It was transformed into a research database by the National Health Research Institutes (NHRI, Chunan, Taiwan). The identification numbers of all individuals in the reimbursement data file were encrypted to protect the patients' privacy. These files contained detailed demographic data (including birth date and gender) and information regarding the health care services provided for each patient, including all payments for outpatient visits, hospitalizations, prescriptions, and intervention procedures. In addition, up to five diagnoses were provided for the hospitalization.

Although the NHI of Taiwan began to facilitate palliative and end-of-life care for noncancer-related terminal cases in 2009, most physicians and health care professionals were unable to differentiate who could be regarded as “terminal,” or expected to live less than 6–12 months. As the law in Taiwan did not allow withdrawing mechanical respirator once it was installed before January of 2011, our team worked with the NHRI to acquire and analyze data before 2010 to avoid underestimation of the survival time for possible withdrawal of MV. Hence, individuals who had undergone invasive or noninvasive respiratory care at least once during the period from 2003 to 2007 were included because of comprehensiveness.

Because the government has established guidelines stating that no more than 10% of all data can be drawn, we extracted a random sample of these patients with a 3.4:1 sampling ratio. Namely, one patient record was randomly drawn from every 3.4 records so that the total amount of data would be less than the 10% limit. Subjects who were older than the age of 17 years and had undergone PMV were enrolled. To select patients receiving MD due to end-stage renal disease, the dataset was linked with the registry of MD in the registration files of catastrophic illness patients of the NHI Research Database.

### Statistical analysis

#### Prognostic factors

Age, gender, hospital size, and comorbidities were included as the major prognostic factors for these patients. Differences in survival were examined across genders and four categories of age (younger than 60, 61–70, 71–80, and 81 years or older). The hospital size was retrieved from the NHI claim codes and was classified into nonmedical center (usually fewer than 1000 beds) or medical center (usually more than 1000 beds). The data for each inpatient hospitalization included up to five diagnoses, which were coded according to The International Classification of Diseases, Ninth Revision, clinical modification (ICD-9 CM). Comorbidities that occurred in <5% of the PMV study cohort were excluded. The comorbidities included are listed in Table 1.

**Table 1.** The Comorbidities and Their Codes Included as the Prognostic Factors of Patients Treated with Prolonged Mechanical Ventilation and Maintenance Dialysis

Comorbidities	ICD-9 codes (diagnoses)
Septicemia	038
Cancer	140–208
DM	250
Anemia	280 (Iron deficiency anemia), 285 (other and unspecified anemia)
Hydrocephalus	331.3 (Communicating hydrocephalus), 331.4 (obstructive hydrocephalus)
Anoxic brain damage	348.1
HTN	401–405
Acute myocardial infarction	410
Ischemic heart disease	411–414
Heart failure	428
Intracranial hemorrhage	430–432
Stroke	433 (Occlusion and stenosis of precerebral arteries)
	434 (Occlusion of cerebral arteries)
Arterial embolism and thrombosis of lower extremity	444.22
Pneumonia	481–486
COPD	490–496
Peptic ulcer	530.2 (Ulcer of esophagus), 531 (gastric ulcer), 532 (duodenal ulcer), 533 (peptic ulcer site unspecified), 534 (gastrojejunal ulcer)
Peritonitis	567 (Peritonitis and retroperitoneal infections)
Cirrhosis	571.2 (Alcoholic cirrhosis of liver), 571.5 (cirrhosis of liver without mention of alcohol)
Gastrointestinal hemorrhage	578
Urinary tract infection	599.0 (Urinary tract infection, site not specified)
Pressure ulcer	707.0
Gangrene	785.4
Shock	785.5 (Shock without mention of trauma)
Device complications	996.1 (Mechanical complication of other vascular device, implant, and graft), 996.6 (infection and inflammatory reaction due to internal prosthetic device implant and graft)

COPD, chronic obstructive pulmonary disease; DM, diabetes mellitus; HTN, hypertension; ICD-9, International Classification of Diseases, Ninth Revision.

Univariate Cox regression analysis was first conducted to examine the association between each prognostic variable and the length of survival. Spearman's rank correlation was used to explore any colinearity between the studied variables. Multivariable Cox regression analyses were performed to investigate the impact of prognostic factors after adjusting for potential confounding variables. Variables considered in the models included gender, four categories of age, hospital size (nonmedical center or medical center), and comorbidities. Backward selection processes were applied to model the comorbidities of prognostic relevance. Comorbidities were considered both individually and concurrently in the Cox regression models. All statistical analyses were performed using SAS version 9.4 software (SAS Institute, Cary, NC).

#### Survival analysis and estimation of life expectancy

Each patient receiving MD who fulfilled the definition of PMV was followed from the 21st day of MV until he or she died or was censored on December 31, 2007. The median survival and long-term survival rates were estimated using the Kaplan–Meier method. Lifetime survival (life expectancy) of the patients (up to 300 months) were obtained using linear extrapolation of a logit-transformed curve of the survival ratio between the PMV and an age- and gender-matched reference population, as generated by the Monte Carlo method from the life table of the general population of Taiwan. Detailed methods and mathematical proof assuming a constant excess hazard can be found in our previous reports.^19,20^

To facilitate computation, we used iSQoL, a software program that was built for the above calculations and can be downloaded for free from the following website: www.stat.sinica.edu.tw/isqol

## Results

### Patient survival, characteristics, and comorbidities

During the study period, there were 30,559 patients receiving PMV. Of which, 1035 patients were receiving MD (Table 2), and 12.1% was censored at the end of 2007. The median length of survival of patients receiving PMV and MD was 54 days. Their mean (±standard deviation) age was 69.3 (±11.3) years, and 52.7% were female. Compared with patients receiving PMV, but not MD, there were more patients who were female (52.7% vs. 39.7%), younger than 70 years old (48% vs. 33.2%), more in a medical center (42.5% vs. 37.3%), less comorbid with chronic obstructive pulmonary disease (COPD; 3.5% vs. 16.6%), cancer (5.9% vs. 12.4%) or pneumonia (38.6% vs. 47.9%), more comorbid with diabetes mellitus (DM), hypertension (HTN), septicemia, shock, peritonitis, and device complications (Table 2).

**Table 2.** Comparison Between Patients Receiving Prolonged Mechanical Ventilation Treated With and Without Maintenance Dialysis Stratified by Clinical Factors

Clinical factors	No. of patients (%)		p^*^
	With MD	Without MD	
Total	1035 (3.4%)	29,524 (96.6%)	
Survival rate			
>30 Days	67.6%	75.3%	
>91 Days	40.8%	55.3%	
>182 Days	24.1%	44.5%	
>365 Days	12.6%	34.4%	
Censored	12.1%	26.8%	
Life expectancy (days, 95% CI)	265 (249–281)	723 (716–731)	<0.0001
Gender female (%)	545 (52.7)	11,711 (39.7)	<0.0001
Age (years)			<0.0001
≤60	218 (21.1%)	5258 (17.8%)	
60–70	278 (26.9%)	4559 (15.4%)	
70–80	392 (37.9%)	10,101 (34.2%)	
>80	147 (14.2%)	9606 (32.5%)	
Hospital size			
Medical center, *N* (%)	440 (42.5)	11,013 (37.3)	0.0007
Chronic comorbidities, *N* (%)			
DM	355 (34.3)	5779 (19.6)	<0.0001
HTN	318 (30.7)	4008 (13.6)	<0.0001
COPD	36 (3.5)	4907 (16.6)	<0.0001
Cancer	61 (5.9)	3669 (12.4)	<0.0001
Urological cancer^a^	15 (1.4)	130 (0.4)	<0.0001
Hydrocephalus	26 (2.5)	969 (3.3)	0.1701
Cirrhosis	26 (2.5)	800 (2.7)	0.7000
Acute comorbidities, *N* (%)			
Pneumonia	399 (38.6)	14,155 (47.9)	<0.0001
Septicemia	319 (30.8)	7091 (24.0)	<0.0001
Shock	190 (18.4)	3662 (12.4)	<0.0001
Gangrene	11 (1.1)	225 (0.8)	0.2774
Peritonitis	32 (3.1)	447 (1.5)	<0.0001
Device complications	64 (6.2)	211 (0.7)	<0.0001

^a^ Urinary bladder or kidney cancer (ICD-9: 188 malignant neoplasm of bladder, 189 malignant neoplasm of kidney and other unspecified urinary organs).

^*^ *p*-Value refers to the comparison between patients receiving PMV treated with and without MD, using the chi-square test.

CI, confidence interval; MD, maintenance dialysis; PMV, prolonged mechanical ventilation.

The most frequently encountered comorbidities were pneumonia (38.6%), followed by DM (34.3%), septicemia (30.8%), HTN (30.7), and shock (18.4%), as summarized in Table 2. Although these patients had a lower prevalence of cancer compared to those without MD, the proportion of urinary bladder or kidney cancer was higher.

**FIG. 1.**
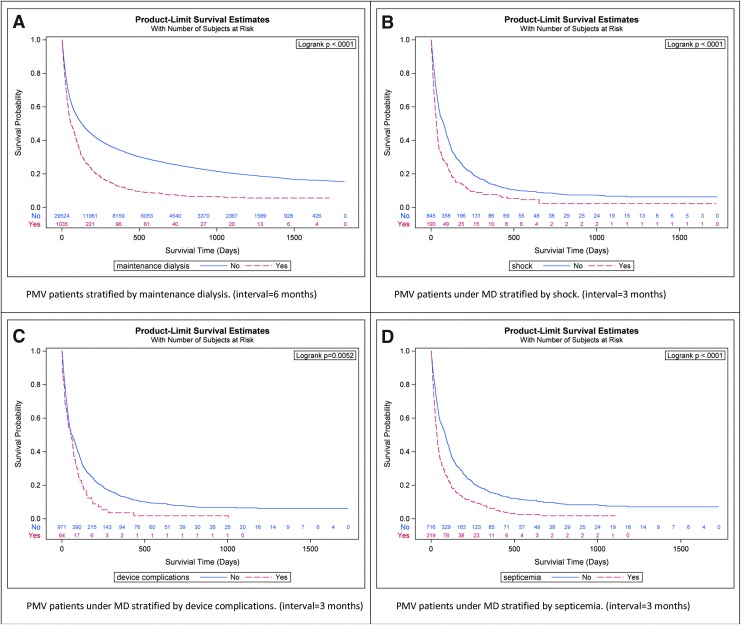
The Kaplan–Meier's estimates of the survival rates of PMV patients stratified by MD **(A)**, PMV patients under MD stratified by shock **(B)**, device complications **(C)**, septicemia **(D)**. MD, maintenance dialysis; PMV, prolonged mechanical ventilation.

The length of survival of patients receiving PMV and MD was much shorter than that of patients receiving PMV but not MD (Table 2 and Fig. 1A). The three-month, six-month, and 1-year survival rates of patients receiving PMV and MD were 40.8%, 24.1%, and 12.6%, respectively (Table 3). The life expectancy was 265 days. Only 12.6% of patients receiving PMV and MD survived more than one year, that is, nearly seven out of eight patients died within one year.

**Table 3.** Survival Rates of Patients Receiving Prolonged Mechanical Ventilation and Maintenance Dialysis at Each Survival Time Stratified by Prognostic Factors

Variable	No. of patients	Survival %				Life expectancy days (95% CI)
		Greater than one month, %	Greater than three months, %	Greater than six months, %	Greater than one year, %	
All	1035	67.6	40.8	24.1	12.6	265 (249–281)
Gender						
Female	545	67.9	44.4	27.4	15.3	371 (343–397)
Male	490	67.3	36.9	20.5	9.6	152 (137–175)
Age (years)						
≤60	218	65.9	46.3	30.6	18.6	454 (396–503)
61–70	278	70.0	41.8	26.6	14.5	278 (245–312)
71–80	392	68.3	38.4	19.9	9.9	152 (134–168)
>80	147	63.9	37.5	20.9	8.0	126 (104–150)
Hospital size						
Medical center	440	66.9	43.2	27.4	17.2	409 (362–452)
Nonmedical center	595	68.2	39.1	21.8	9.5	147 (133–163)
Chronic comorbidities						
Cancer						
No	974	68.7	42.0	24.8	12.8	261 (242–279)
Yes	61	49.8	21.8	12.3	10.1	112 (80–139)
Cirrhosis						
No	1009	67.7	41.2	24.4	12.9	
Yes	26	65.4	24.5	9.8	0	
Hydrocephalus						
No	1009	68.0	41.0	24.3	12.8	
Yes	26	53.9	34.6	18.3	4.6	
Acute comorbidities						
Septicemia						
No	716	73.2	47.5	28.8	15.4	357 (332–388)
Yes	319	55.1	26.0	13.7	6.3	90 (79–104)
Shock						
No	845	70.8	44.2	26.4	13.8	299 (281–317)
Yes	190	53.7	26.2	14.2	7.4	108 (85–131)
Device complications						
No	971	68.0	41.5	24.9	13.2	298 (275–315)
Yes	64	62.3	30.7	10.9	3.6	81 (62–98)
Peritonitis						
No	1003	68.0	41.5	24.7	12.9	
Yes	32	56.3	18.8	6.3	3.1	
Gangrene						
No	1024	67.9	41.0	24.2	12.8	
Yes	11	45.5	27.3	18.2	0	

### Survival rates and life expectancies of patients receiving PMV and MD stratified by characteristics or comorbidities

Patients who were male or older than the age of 70 had poorer prognosis (Table 3). Less than 10% of the male patients or patients older than the age of 70 could survive over one year. Patients with major chronic and/or acute comorbidities usually survived even shorter periods. Less than 5% of patients with comorbidities such as cirrhosis, hydrocephalus, device complications, peritonitis, or gangrene could survive over one year. Less than 10% of patients comorbid with cirrhosis or peritonitis could survive over six months. Patients comorbid with shock had an estimated life expectancy of less than four months (Fig. 1B), while those with comorbid device complications or septicemia had an estimated life expectancy of less than three months (Fig. 1C, D).

### Prognostic factors identified by Cox's regression model

The results of multivariable Cox regression models are summarized in Table 4. Patients who were male, older than 80 years, in a nonmedical center, or with any comorbidity of septicemia, shock, device complications, peritonitis, gangrene, cancer, cirrhosis, and hydrocephalus showed an adjusted hazard ratio significantly >1. This magnitude of effects was the same or robust under both strategies of stepwise and backward selection. After control of potential confounding by other variables, patients treated in medical centers showed a slightly better prognosis. In contrast, male gender, old age, comorbid with cancer, cirrhosis, hydrocephalus, septicemia, shock, device complications, peritonitis, and gangrene predicted a shorter length of survival. Among chronic comorbidities, the adjusted hazard ratio of cirrhosis was higher than that of cancer. Among acute comorbidities, the adjusted hazard ratios of peritonitis and gangrene were the highest.

**Table 4.** Estimates of Hazard Ratio and 95% Confidence Interval of Proportional Hazards Model for Patients Treated with Both Prolonged Mechanical Ventilation and Maintenance Dialysis According to Risk Factors

Variables	No. (total = 1035)	Crude HR (95% CI)	Adjusted HR*^a^*(95% CI)	*p*-Value aHR
Male/female	545/490	1.22 (1.07–1.39)	1.21 (1.06–1.39)	0.005
Age (years old)				
61–70/18–60	278/218	1.10 (0.91–1.34)	1.12 (0.92–1.36)	0.269
71–80/18–60	392/218	1.28 (1.07–1.53)	1.19 (0.99–1.43)	0.067
≥81/18–60	147/218	1.38 (1.11–1.73)	1.28 (1.01–1.60)	0.038
Medical center (yes/no)	440/595	1.22 (1.07–1.39)	1.18 (1.03–1.36)	0.019
Chronic comorbidities				
DM (yes/no)	355/680	0.89 (0.77–1.02)		
HTN (yes/no)	318/717	0.90 (0.78–1.04)		
Cancer (yes/no)	61/974	1.47 (1.12–1.94)	1.67 (1.25–2.22)	<0.001
Cirrhosis (yes/no)	26/1009	1.53 (1.02–2.30)	1.71 (1.13–2.59)	0.011
Hydrocephalus (yes/no)	26/1009	1.36 (0.91–2.02)	1.50 (1.00–2.26)	0.050
Acute comorbidities				
Pneumonia (yes/no)	399/636	1.04 (0.91–1.19)		
Septicemia (yes/no)	319/716	1.68 (1.46–1.94)	1.51 (1.29–1.76)	<0.001
Shock (yes/no)	190/845	1.59 (1.35–1.87)	1.34 (1.12–1.61)	0.002
Device complications (yes/no)	64/971	1.45 (1.11–1.88)	1.48 (1.13–1.92)	0.004
Peritonitis (yes/no)	32/1003	1.74 (1.21–2.49)	1.84 (1.28–2.66)	0.001
Gangrene (yes/no)	11/1024	1.58 (0.87–2.87)	1.98 (1.08–3.63)	0.027

^a^ Adjusted hazard ratio is the hazard ratio adjusted for other variables by backward selection processes.

HR, hazard ratio.

## Discussion

Although there are studies reporting the poor prognosis of patients under PMV,^9,21,22^ to the best of our knowledge, we are among the first to explore the crucial estimates of detailed survival rates and life expectancies for patients receiving both PMV and MD based on a national database, as summarized in Tables 3 and 4. The prognosis of these patients were generally poor, as they usually suffered from additional comorbidities of DM, HTN, urological cancer,^23^ device complications,^24^ and peritonitis.^25^ In this study, our national sample showed that the life expectancy of patients receiving PMV but not MD would be less than two years, and only 34.4% of these patients could survive for more than one year, that is, nearly two of three patients would die within one year (Table 2).

In contrast, patients receiving PMV and MD would die even earlier. Among them, life expectancies of such patients with any of the major comorbidities were consistently less than nine months (Table 3). Without additional risk factors, over three-quarters of the patients fulfilled the definition of terminal illness used by Medicare, that is, a survival time of less than six months.^26^ In addition, only 12.6% could survive over one year (Table 2). Namely, 87.4% fulfilled the definition of terminal illness used by the General Medical Council and the National Institute for Health and Care Excellence (NICE), that is, survival time of less than one year.^27,28^ Moreover, if such patients were comorbid with cancer, cirrhosis, septicemia, shock, device complications, or peritonitis, the prognosis is even poorer with shorter life expectancies (Table 3 and Fig. 1).

Before we make further inference, however, we must provide arguments to validate the accuracy of our estimation. First, because our patients were selected by 1:3.4 systematic random sampling from the national population, the representativeness of this study would generally be assured. Compared to patients receiving PMV but not MD, patients receiving both PMV and MD were found to have higher proportions of urinary bladder and kidney cancer (Table 2), which is compatible with previous reports and corroborates the above claim.^29,30^ Second, the longest life expectancy among the different subcohorts younger than 60 years of age was only 454 days (Table 3). Among our sample, only 12.1% of the patients under both PMV and MD were censored. Therefore, the five-year follow-up period from 2003 to 2007 would generally be adequate for the estimation of life expectancy. Third, patients with risk factors of shorter life expectancy in Table 3 were cross-validated by the results of multivariable proportional hazards model in Table 4. We thus tentatively conclude that these estimates would be relatively accurate and useful for medical decision making. Palliative care is important and early referral for end-of-life care is recommended for these patients, especially those with major comorbidities.

Compared with patients receiving PMV but not MD, a lower proportion of patients receiving PMV and MD had cancer (5.9% vs. 12.4%) or COPD (3.5% vs. 16.6%); the age distribution of patients receiving PMV and MD was younger and nearly half were <70 years old, and more received care in a medical center, that is, better equipped with expensive medical technology and qualified professionals (Table 2). These characteristics might provide additional uncertainty or difficulty in the decision making of whether or not to withhold cardiopulmonary resuscitation or allow natural death.

A previous study found that family members and care-givers were more likely than clinicians to expect the patient to stay alive.^18^ Patient characteristics of younger than 70 years old, lower proportions with cancer or COPD, and higher proportions in medical centers may possibly bring the hope of successful weaning to family or care-givers. If the patient died earlier than the family members anticipated, this might make the family more depressed or sad. Prognostication and timely recognition of death can facilitate patients and/or families to discuss and prepare for the end of life in advance. Therefore, this study on the factors predicting survival may provide important information to family and/or patients and avoid prolonged suffering during the process (Table 3).

The multivariable proportional hazards model corroborates the above results and consistently showed independent significant factors, including gender, age, hospital size, septicemia, shock, device complications, peritonitis, gangrene, cancer, cirrhosis, or hydrocephalus (Table 4). Compared with other prediction models, our results seem easier for memorization and application at bedside. Based on the characteristics and diagnosis when admitted for PMV and MD, we would be able to find out which patients fulfilled the definition of terminal illness. Because the model is an exponential one, the presence of two risk factors would be multiplicative. In other words, the presence of more than two risk factors indicates a very poor prognosis by multiplying the hazard ratios of both of them. End-of-life care should be considered at an even earlier stage for these patients.

## Limitations

Our study has at least the following limitations. First, to fulfill all the reimbursement regulations of the NHI, it is possible that some recorded diagnoses were overrepresented because they were reimbursed more easily. However, the NHI of Taiwan has offered a list of 30 major categories of catastrophic illnesses that are exempt from partial copayments,^31^ and each has its specific diagnostic criteria to prevent any abuse. For example, no types of malignant neoplasms require copayments, and evidence of histopathology and/or cytology is generally required for diagnoses of cancer, except hepatocellular carcinoma. In addition, a diagnosis of end-stage renal disease requires MD with documentation of chronic kidney disease with an irreversible creatinine level >8 mg/dL, or creatinine level >6 mg/dL with DM as a comorbid condition.^32^

Second, because there were only five diagnoses that could be included in the database, the effect of some diagnoses indirectly related to the death of patients might be underestimated. However, presence of such illnesses would generally aggravate the patient's condition. Namely, the prediction of our model would be a conservative or optimistic estimate, and would not overestimate the likelihood of mortality for a patient. Thus, referral of such patients to palliative care or end-of-life care would be a reasonable one.

Third, the database did not contain any information regarding the severity of risk factors for different diagnoses. We were, therefore, unable to further stratify patients with the severity factors. However, patients and their families could look at the factors in Table 3 showing a high mortality rate ( = 1-survival rate) within six months or one year as a guide for making decisions. Moreover, if such a patient is also comorbid with a specific diagnosis listed on Table 4 with a high adjusted hazard ratio (aHR) (e.g., cirrhosis), we could refer this patient for end-of-life care earlier.

Fourth, the database did not contain any information regarding the consciousness of patients, and we were thus unable to provide more information on the duration of the patients' comatose states. When making decisions about end-of-life care, this is an important criterion which should be collected in future studies and included in the Cox model for a better understanding of the prognostic factors and thus better decision making.

Fifth, because the retrieved data covered 2003–2007 that were more than a decade ago, the generalizability would be limited. However, since Taiwan began to allow withdrawal of mechanical respiration in January of 2011, our data and analysis would less likely underestimate the survival of these groups of people.

## Conclusion/Implications

In our study, we found that more than 85% of patients receiving PMV and MD would die within one year and more than three-quarters of patients receiving PMV and MD survived less than six months, which largely fulfilled the definitions of terminal illness used by NICE or Medicare. If patients were comorbid with peritonitis or cirrhosis, <10% would survive past six months. Any one of other major comorbidities, including septicemia, shock, device complications, gangrene, cancer, or hydrocephalus, could independently affect their survival, and patients and their families should be informed of this to make decisions for early referral of palliative and/or end-of-life care. Moreover, advance medical directive had better be recommended to patients under either PMV or MD when they first come across one organ system failure.
